# Period2 Deficiency Blunts Hypoxia-Induced Mobilization and Function of Endothelial Progenitor Cells

**DOI:** 10.1371/journal.pone.0108806

**Published:** 2014-09-30

**Authors:** Tao Qin, Yuan-Yuan Sun, Wen-Wu Bai, Bo Wang, Yi-Fan Xing, Yan Liu, Rui-Xue Yang, Yu-Xia Zhao, Jian-Min Li

**Affiliations:** 1 Key Laboratory of Cardiovascular Remodeling and Function Research, Chinese Ministry of Education and Chinese Ministry of Health, Shandong University, Jinan, Shandong, China; 2 Department of Traditional Chinese Medicine, Qilu Hospital, Shandong University, Jinan, Shandong, China; 3 Department of Emergency Surgery, Qilu Hospital, Shandong University, Jinan, Shandong, China; 4 Department of Orthopedics, Qilu Hospital, Shandong University, Jinan, Shandong, China; European Institute of Oncology, Italy

## Abstract

**Background:**

In the clinic, variations in circadian rhythm are evident in patients with cardiovascular disease, and the risk of cardiovascular events increases when rhythms are disrupted. In this study, we focused on the role of the circadian gene period2 (per2) in mobilization and function of endothelial progenitor cells (EPCs) *in vitro* and *in vivo* after myocardial infarction (MI) in mice.

**Methods and Results:**

MI was produced by surgical ligation of the left anterior descending coronary artery in mice with and without per2 deficiency. Trans-thoracic echocardiography was used to evaluate cardiac function in mice. Per2^−/−^ mice with MI showed decreased cardiac function and increased infarct size. The number of CD34+ cells and capillary density were decreased in the myocardium of per2^−/−^ mice on immunohistochemistry. Flow cytometry revealed decreased number of circulating EPCs in per2^−/−^ mice after MI. *In vitro*, per2^−/−^ EPCs showed decreased migration and tube formation capacity under hypoxia. Western blot analysis revealed inhibited activation of extracellular signal-regulated kinase and Akt signaling in the bone marrow of per2^−/−^ mice and inhibited PI3K/Akt expression in per2^−/−^ EPCs under hypoxia.

**Conclusions:**

Per2 modulates EPC mobilization and function after MI, which is important to recovery after MI in mice.

## Introduction

For most of the past 2 decades, the role of the microvasculature in recovery from myocardial infarction (MI) has been considered important. Studies of humans and animals have shown that a subpopulation of mononuclear cells with enhanced potential for differentiation into endothelial cells are mobilized endogenously from the bone marrow in response to MI and become incorporated into sites of new vessel growth in the ischemic tissue [1]–[4]. These cells are known as endothelial progenitor cells (EPCs). Circulating EPC level and function are predictive of prognosis following acute MI and are associated with cumulative cardiovascular risk, cardiovascular mortality and atherosclerosis progression in patients with coronary artery disease [5]–[11]. Mobilization of CD34+ cells by drugs or transplantation of *ex vivo*-expanded EPCs could improve cardiac function after myocardial ischemia [12], [13], [35].

Variations in circadian rhythm are evident in patients with cardiovascular diseases, and the risk of cardiovascular events increases when rhythms are disrupted [14]–[17]. Epidemiologic studies have demonstrated circadian patterns associated with the incidence of cardiovascular disease. For example, the onset of MI is markedly increased between 6:00 AM and 12:00 PM [18]. However, the specific relationship between a circadian gene and mobilization of bone-marrow EPCs associated with early MI has not been determined.

Period2 (per2) regulating EPC function under hypoxia has not been determined. Given that circadian rhythms control the cell cycle and tumor growth [19], per2 may be able to alter the response of bone marrow and EPC function to ischemic injury. Therefore, we investigated the effect of per2 deletion on the response of bone-marrow EPC mobilization and function in mice with MI and *in vitro*.

## Methods

### Mouse Model of MI

C57BL/6 wild-type (WT) mice (n = 30, 8–12 weeks old) weighing 25–30 g were obtained from VITAL RIVER (Beijing). Per2^−/−^ mice (n = 25) were obtained from the Model Animal Research Center, Nanjing University (Nanjing, China), and backcrossed for more than 10 generations onto a C57BL/6 inbred background. All animal studies were carried out at the Animal Care Center of the Key Laboratory of Cardiovascular Remodeling and Function Research, Shandong University (Shandong, China). The experiment followed the Animal Management Rule of the Ministry of Public Health, People's Republic of China (document no. 55, 2001), and the experimental protocol was approved by the Animal Care Committee of Shandong University.

Mice were acclimatized in the same room with a 12-h/12-h light–dark cycle for at least 2 weeks before experiments. MI was induced by surgical ligation of the left anterior descending (LAD) coronary artery as described [20]. Mice were anesthetized with sodium pentobarbital (50 mg/kg), then the chest was opened at the left fourth intercostal space and the LAD was ligated with a 7–0 silk suture. Successful ligation was verified by color change in the artery.

### Assessment of Cardiac Function

Four weeks after MI, trans-thoracic echocardiography (Visual Sonics Vevo 770, Canada) was used to evaluate cardiac function in mice. We measured the left-ventricular (LV) internal dimension diastolic, LV internal dimension systolic, systolic ejection fraction and percent LV fractional shortening (FS). An observer blinded to the experiment performed the measurements for at least 3 consecutive pulsation cycles.

### Histology

Briefly, mice were euthanized with sodium pentobarbital (50 mg/kg). The chest was opened and the heart was arrested in diastole by intraventricular injection of KCL (10%). The myocardial vasculature was perfused with 4% formalin for 10 min. Hearts were harvested and fixed in 4% formalin for 48 hr. Cardiac fibrosis was assessed by Masson's trichrome staining. The infarct size and fibrosis areas were expressed as the sum of the epicardial and endocardial scar length divided by the sum of the LV epicardial and endocardial circumferences [21]. CD34 (1∶200, Abcam, USA) immunohistochemistry was used to assess EPCs in the infarcted myocardium. Capillary density in the peri-infarct area was determined 28 days after infarction. Paraffinized LV 5-µm sections were immunohistochemically stained with anti-CD31 monoclonal antibody (PECAM-1, 1∶50, R&D, Germany). Briefly, sections were deparaffinized, pretreated with 0.3% H_2_O_2_ for 20 min to inhibit endogenous peroxidase activity, then blocked with 2% goat serum for 30 min and incubated with primary antibody overnight at 4°C. Visualization involved the avidinbiotin-complex technique and high-sensitivity diaminobenzidine (DAB+) chromogenic substrate system (Dako Denmark), then counterstaining with hematoxylin. We counted 6 randomly selected 400×fields, and mean CD34+ or CD31+ cells per field was obtained for statistical analysis.

### Evaluation of Circulating EPCs

Circulating EPCs were quantified 3 days after the onset of MI. Circulating EPCs were defined as CD34+ (FITC-conjugated anti-mouse CD34 antibody, eBioscience) and positive for endothelial-specific antigen KDR (APC-conjugated anti-mouse KDR antibody, eBioscience) [22]–[24]. FITC- or APC-conjugated isotype IgG antibody (eBioscience) was used as a control. Cells were analyzed by use of a fluorescence-activated cell sorter (FACS caliber, BD Biosciences) and CD34+ and KDR+ EPCs were expressed as proportion of mononuclear cells.

### Bone-marrow Cell Isolation and EPC Culture

Hollow bones of mouse legs were extracted by standard surgical procedures, and whole bone marrow was harvested by flushing the marrow out with 500 µl phosphate buffered solution (PBS) by use of a syringe with a 20-gauge needle. Some bone marrow extracts were shock-frozen before analysis.

EPC isolation, *ex vivo* expansion and culture of EPCs was performed as previously described [25]. In brief, bone-marrow mononuclear cells were isolated from mice by density-gradient centrifugation with lymphocyte separation medium (mouse) (Solarbio, China). Cells were plated on culture dishes coated with mice vitronectin (Sigma) and cultured in phenol red–free endothelial-cell basal medium-2 (Lonza, Germany) supplemented with hydrocortisone, human fibroblast growth factor B, vascular endothelial growth factor (VEGF), R3 insulin-like growth factor 1, ascorbic acid, human endothelial growth factor, GA-1000, heparin and 5% fetal bovine serum. Cells were maintained at 37°C with 5% CO_2_ in a humidified atmosphere for 4 days, then non-adherent cells were removed by washing with PBS and new medium was added. The culture was maintained through day 7, when EPCs were recognized as attached spindle-shaped cells. Cells were extensively washed with PBS, and adherent cells were incubated with 2.4 µg/ml 1,1′-dioctadecyl-3,3,3′,3′-tetramethylindocarbocyanine perchlorate-acetylated low-density lipoprotein (DiI-Ac-LDL, Invitrogen) and stained with FITC-labelled Ulex europaeus agglutinin 1 (lectin, 10 µg/ml; Sigma) for EPCs. EPCs were also characterized by immunofluorescence staining for the expression of VEGF receptor 2 (Flk1, Abcam), platelet/endothelial cell adhesion molecule-1 (CD31, R&D), and CD34 (Abcam). The fluorescent images were recorded under a laser scanning confocal microscope.

In some experiments, 7-day EPCs from WT and per2^−/−^ mice were incubated in a hypoxic condition (1% O_2_) for 24 hr.

### EPC Migration and Tube Formation

Migration of EPCs was investigated under normoxic and hypoxic conditions with the modified Boyden chamber assay as described [26]. In brief, 2×10^4^ EPCs were cultured in inlets (Costar, 8-µm pore size) placed in 24-well culture dishes containing 500 µl endothelial basal medium (Lonza, Germany) and 50 ng/ml VEGF (Prospec, Ness Zina, Israel). After 24 hr, migrated cells were fixed with 4% paraformaldehyde (Sigma) in PBS for 30 min, then wiped gently with a cotton ball to remove non-migratory cells and stained with 2% crystal violet in ethanol. Data are presented as mean number of migrated cells in 5 randomly selected fields at 200× magnification in every membrane (n = 6 in each group).

For tube-formation ability, we used the matrix gel tube formation assay. First, 50 µl matrix gel was added into every well of 96-well plates at 37°C for 1 hr. An amount of 2×10^4^ EPCs supplemented with 50 µl EBM-2 with VEGF (50 ng/ml) was placed onto the matrix gel. The plate was placed under hypoxic conditions for 24 hr. Data are shown as the mean tube number of 5 randomly selected spaces at 400× magnification in each well. Every well was studied at least 3 times (n = 6 in each group).

### Western Blot Analysis

Bone-marrow extracts or cell lysates from EPCs were mixed with sample loading buffer and separated under reducing conditions on 10% SDS-polyacrylamide gel, then incubated with the antibody rabbit anti-per2 (Santa Cruz Biotechnology), anti-PI3k, rabbit anti-Akt or anti-phosphorylated-Akt (p-Akt, pSer473), or rabbit anti-ERK or p-ERK (all Cell Signaling Technology). Protein and phosphorylation levels were normalized to that of β-actin (mouse-antiβ-actin, Kangchen Biotech) and baseline expression.

### Statistical Analysis

Data are expressed as mean ± SEM. SPSS for Windows v16.0 (SPSS Inc., Chicago, IL, USA) was used for statistical analysis. Comparisons between 2 groups involved Student's *t* test and more than 2 groups, one-way ANOVA, followed by least significant difference test (with equal variances assumed) or Dunnett's T3 test (with equal variances not assumed). P<0.05 was considered statistically significant.

## Results

### Per2 deficiency decreased cardiac function and increased infarct size in mice with MI

**Figure 1:**
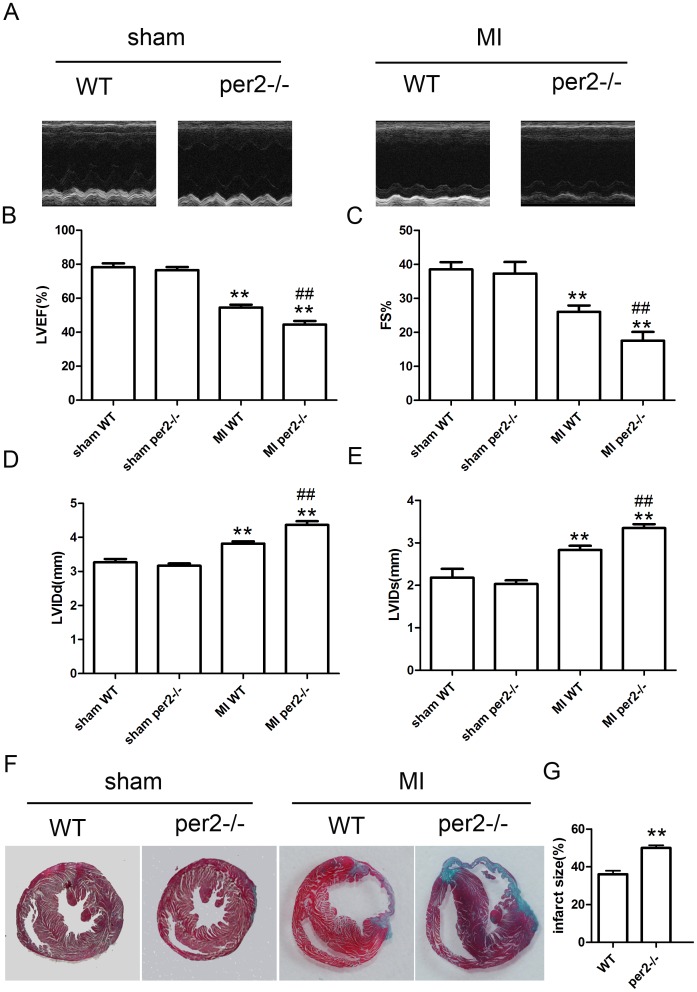
Left ventricular remodeling and infarct size in wild-type (WT) and Period 2-deficient (per2^−/−^) mice after myocardial infarction (MI). (A) Representative M-mode images of papillary muscles. (B–E) Systolic dysfunction 4 weeks after acute MI in mice (** p<0.01 vs sham-operated, ## p<0.01 vs MI WT). (F) Representative Masson's trichrome staining at 28 days after MI. (G) Quantitative analysis of infarct area (** p<0.01 vs WT). LVEF, left-ventricular ejection fracton; FS, fractional shortening; LVIDd, LV internal dimension diastolic; LVIDs, LV internal dimension systolic.

We surgically induced myocardial ischemia in per2^−/−^ and background-matched WT mice. At 4 weeks after MI, mice showed decreased cardiac function (Fig. 1A–E), with cardiac function worse for per2^−/−^ than WT mice. Infarct size was larger for per2^−/−^ than WT mice (Fig. 1F–G).

### Per2 deficiency decreased the number of CD34+ cells and capillary density in the myocardium 4 weeks after MI

**Figure 2:**
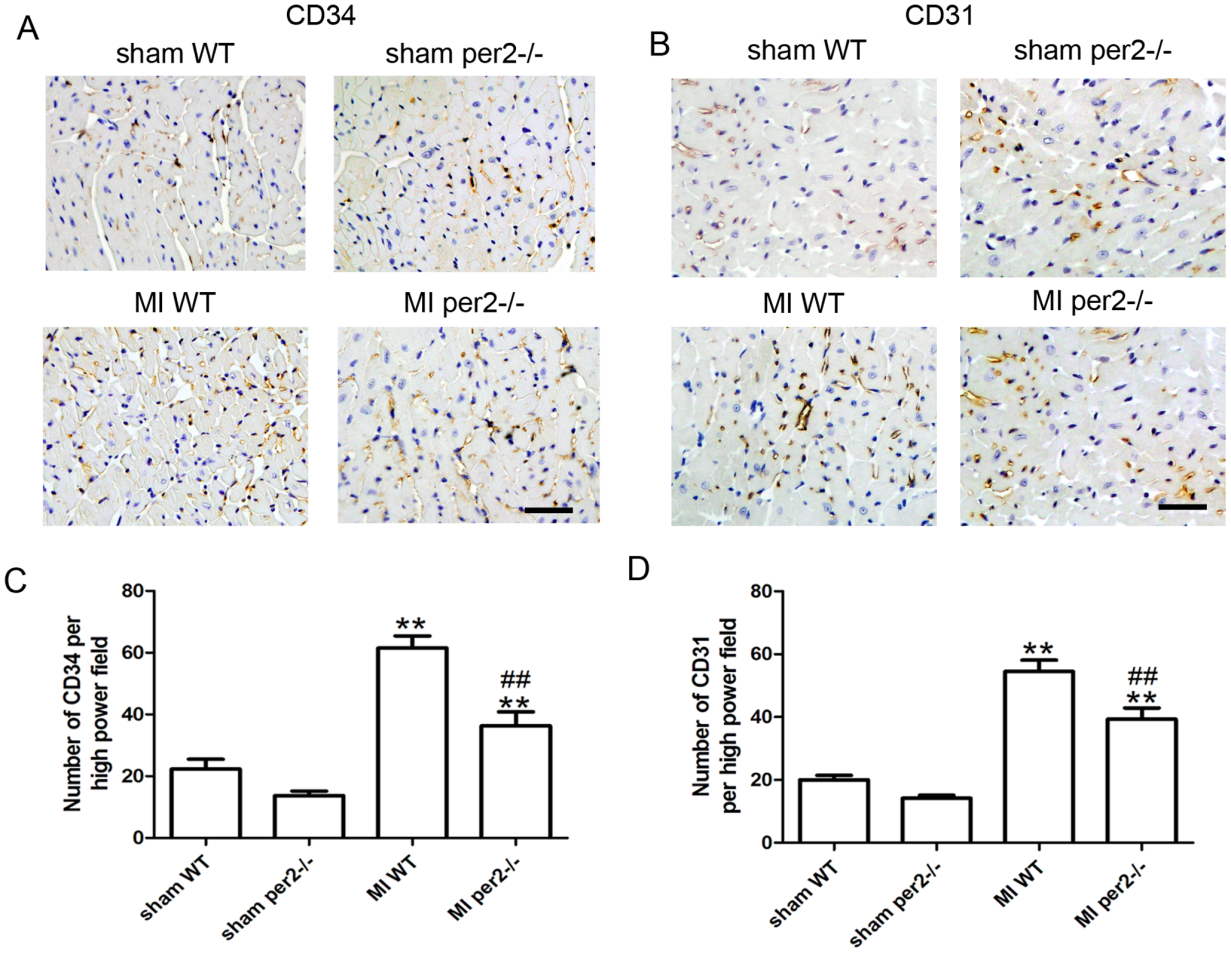
Per2^−/−^ decreased the number of CD34+ progenitors and capillary density in mice. (A) Representative immunostaining of CD34 to identify progenitors and (B) CD31 to identify capillaries. Original magnification: 400×. Quantitative analysis of (C) CD34+ cells and (D) capillary density (** p<0.01 vs sham-operated, ## p<0.01 vs MI WT).

Because EPCs contribute to angiogenesis [1]–[4], we compared the number of CD34+ cells and capillary density in the ischemic heart in WT and per2^−/−^ mice. CD34+ cells and capillary density was lower in per2^−/−^ than WT mice at 4 weeks after MI (Fig. 2). Per2^−/−^ increased the MI area and decreased heart function in part by decreasing EPC angiogenesis.

### Per2 deficiency inhibits ischemia-induced EPC mobilization

**Figure 3:**
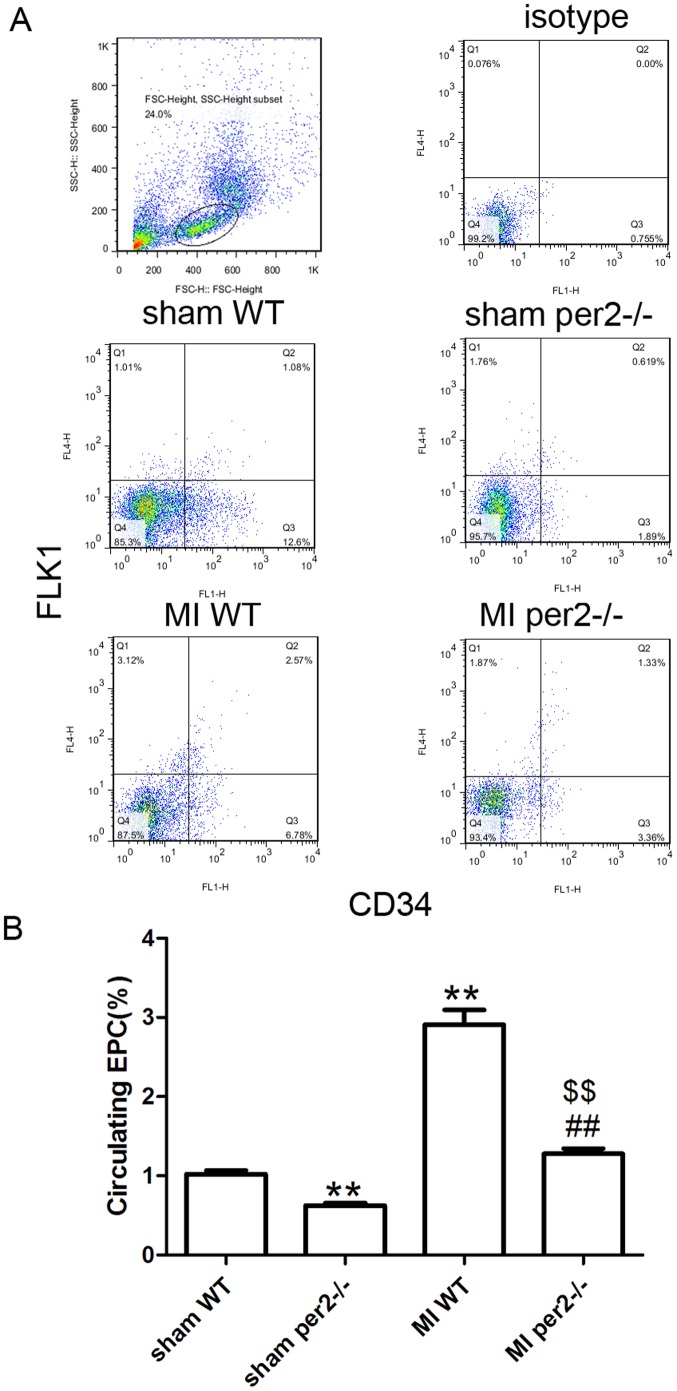
Per2^−/−^ decreased the number of CD34+ Flk-1+ progenitors in blood of mice before and after MI. (A) Representative flow cytometry data of CD34+ Flk-1+ cells, considered EPCs, within the mononuclear cell population. (B) Quantitative evaluation of EPCs (** p<0.01 vs sham-operated WT, ## p<0.01 vs sham-operated per2−/−, $$ p<0.01 vs MI WT).

Because angiogenesis after MI depends in part on EPC mobilization, we investigated the effect of per2 on EPC mobilization in response to tissue ischemia by determining CD34+ Flk-1+ cells in peripheral blood by flow cytometry. Basal circulating EPC number was lower in per2^−/−^ than WT mice (Fig. 3A and B). Consistent with previous findings that tissue ischemia can induce EPC mobilization [28], EPC mobilization was enhanced 3 days after MI in WT mice. In WT mice, the EPC level was increased nearly three-fold that in sham-operated mice after MI (Fig. 3A and B) but in per2^−/−^ mice was increased two-fold that in sham-operated mice, which suggests that per2^−/−^ inhibited bone-marrow EPC mobilization after MI.

### Per2 deficiency inhibited bone-marrow EPC mobilization 3 days after MI by inhibiting Akt and ERK signaling

**Figure 4:**
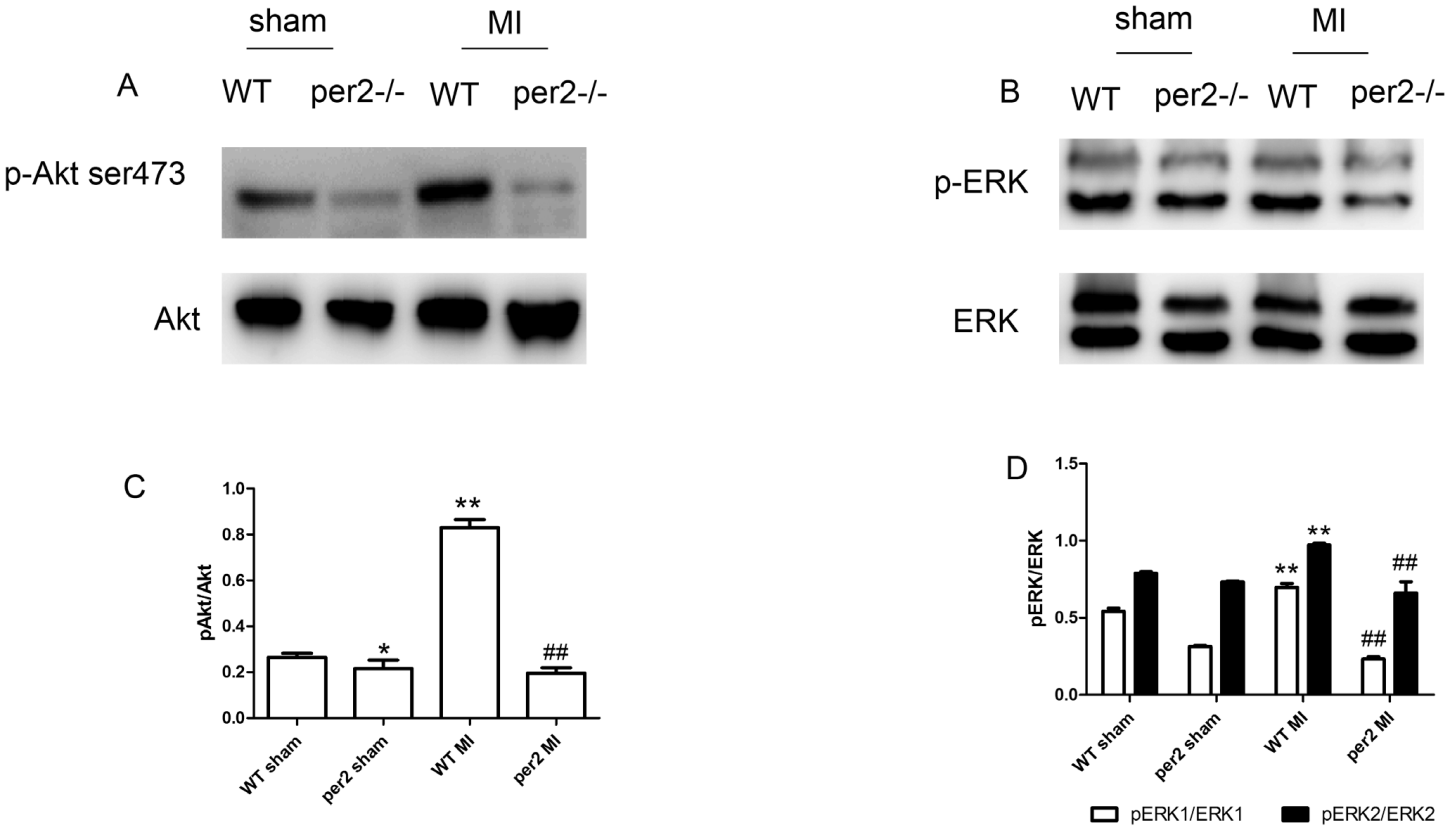
Effect of acute MI on expression of Akt and ERK in mouse bone marrow. Western blot analysis of phosphorylated and total (A) Akt and (B) ERK levels. Quantitative analysis of (C) p-Akt (* p<0.05, ** p<0.01 vs WT sham-operated, ## p<0.01 vs WT MI) and (D) p-ERK (** p<0.01 vs WT sham-operated, ## p<0.01 vs WT MI).

We evaluated the possible mechanisms of per2 affecting bone-marrow EPC mobilization. At 3 days after MI, the bone-marrow environment was changed. Phosphorated Akt and ERK levels were increased both in WT and per2^−/−^ mice, with weaker Akt and ERK phosphorylation in per2^−/−^ bone marrow (Fig. 4A–D).

### Characterization of bone-marrow–derived EPCs

**Figure 5:**
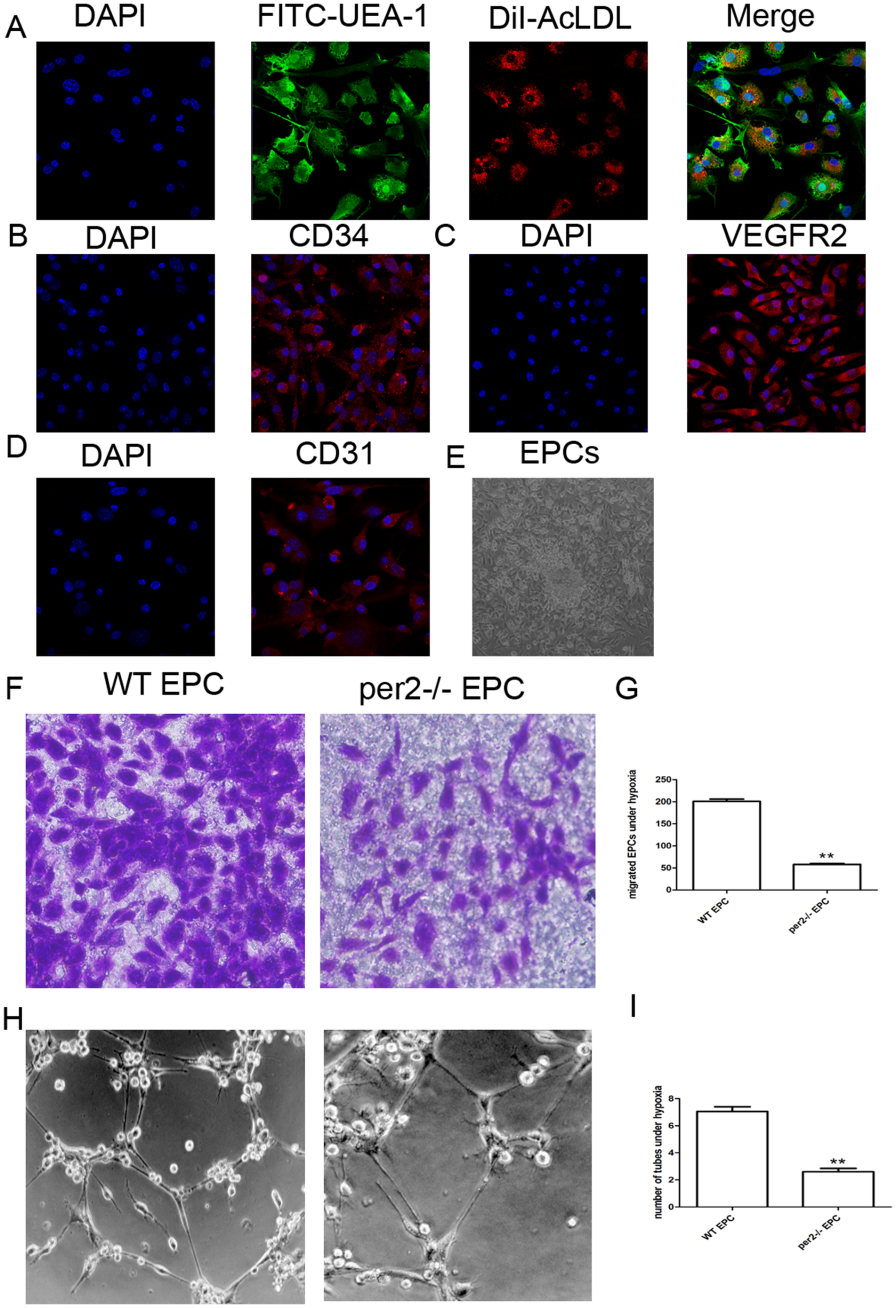
Characterization of cultured EPCs and the effect of per2 on EPC function under hypoxia. (A) Fluorescence microscopy of adherent cells at day 7 after bone-marrow isolation with Dil-Ac-LDL staining and endothelial-specific lectin-FITC binding. (B) The expression of CD34, (C) vascular endothelial growth factor receptor 2 (Flk-1) and (D) CD31 in EPCs. (E) EPCs with phase-contrast imaging. (F) EPC migration evaluated by transwell assay under hypoxia. (G) Quantitative analysis of EPC migration (** p<0.01 s WT EPCs). (H) Tube formation of EPCs detected by matrigel tube-formation assay. (I) Quantitative analysis of EPC tube formation (** p<0.01 vs WT EPCs).

Early EPCs were isolated from bone-marrow mononuclear cells of WT and per2^−/−^ mice. In brief, after 7 days of culture in the presence of endothelial growth medium,>90% of cells were capable of cellular uptake of DiI-AcLDL and FITC-UEA-1 binding (Fig. 5A). Most of the cells expressed CD34 and Flk1 and some expressed CD31 (Fig. 5B–D). EPCs with phase contrast imaging is shown in Fig. 5E.

### Per2 deficiency inhibited EPC function induced by hypoxia

WT or per2^−/−^ EPCs were cultured under normal or hypoxia environment for 24 hr. Hypoxia upregulated EPC function. As compared with WT EPCs, per2^−/−^ EPCs showed weaker migration and tube formation under hypoxia (Fig. 5F–I).

### Per2 deficiency decreased PI3K and phosphorylated Akt levels induced by hypoxia in EPCs

**Figure 6:**
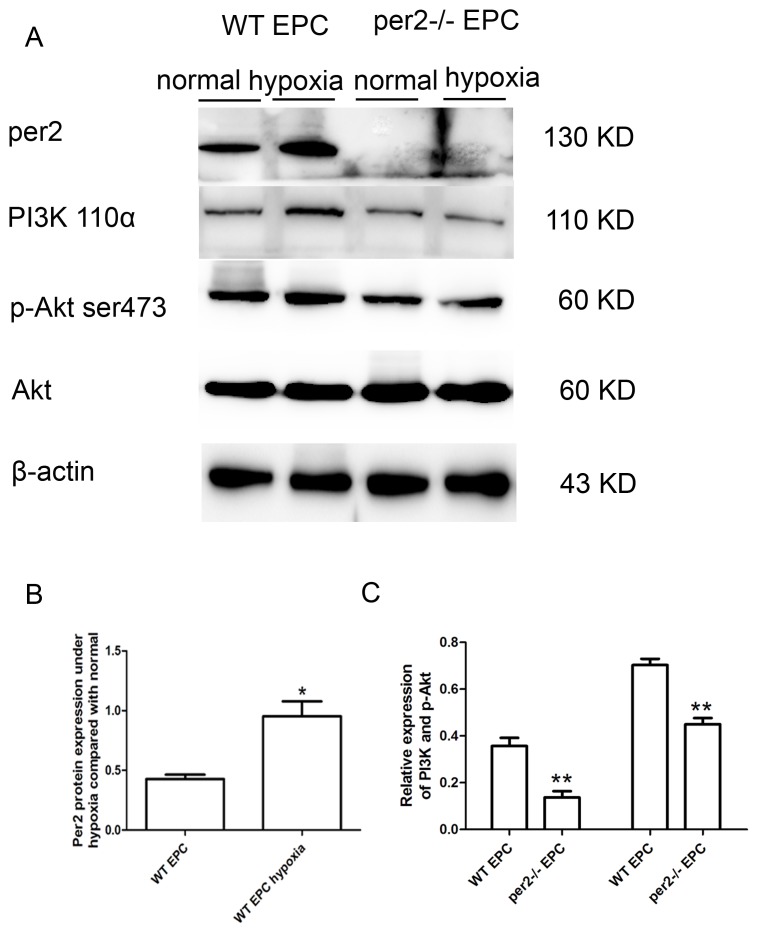
Per2^−/−^ decreased the expression of PI3K and Akt in EPCs under hypoxia. (A) Hypoxia induced the expression of per2 and per2^−/−^ decreased the expression of PI3K and Akt under hypoxia. Quantitative analysis of (B) per2 (* p<0.05 vs WT EPCs), (C) PI3K and p-Akt expression under hypoxia (** p<0.01 vs WT EPCs).

To investigate the mechanism of per2 affecting EPC function under hypoxia, we studied per2 protein level in WT and per2^−/−^ EPCs. In WT EPCs, per2 protein level was increased with hypoxia (Fig. 6A and B). We investigated EPC function-related signaling and found activated PI3K/Akt signaling in WT EPCs under hypoxia (Fig. 6A and C), with reduced signaling in per2^−/−^ mice EPCs.

## Discussion

In this study, we focused on the role of the circadian gene per2 in mobilization and function of EPCs under hypoxia *in vitro* and after MI in mice. Per2^−/−^ mice with MI showed attenuated heart function, increased MI area and decreased angiogenesis. In addition, per2^−/−^ mice showed down regulated Akt and ERK expression in bone marrow, which reduced the mobilization of EPCs in response to acute ischemia. *In vitro* studies further demonstrated that per2^−/−^ suppressed EPC migration and tube-forming capacity under a hypoxic microenvironment. Hypoxia increased the expression of per2 in WT EPCs, which activated PI3K/Akt signaling but was counteracted by per2 knockout. Per2 may play an important role in regulating EPC mobilization and EPC function activity after MI, which is important for recovery.

Ischemic tissue neovascularization requires not just angiogenesis but also circulating EPCs during vasculogenesis [27]. These circulating EPCs are derived from bone marrow and are mobilized in response to tissue ischemia [27]. An inadequate angiogenic response to ischemia in the myocardium of patients might result in poor collateral formation and severe organ damage [28]–[30].

EPCs were recognized to have dual profiles of immature cells, stem or progenitor cells and endothelial-lineage cells in terms of marker expression from peripheral blood or bone marrow mononuclear cells: CD34/Flk-1[31], Sca-1/Flk-1[32], CXCR4/Flk-1 [33], and Flk-1/VE-cadherin [34]. However, the cell-surface marker-based definition of EPCs is still controversial. Here, we analyzed CD34+ Flk1+ cells as a marker of circulating EPCs. Before and after MI, the number of CD34+ Flk1+ EPCs was lower in per2^−/−^ than WT mouse blood. In our previous study [35], we found weaker proliferation of per2^−/−^ than WT EPCs, which explains the decreased number of EPCs before MI.

Evidence from animal studies has been reproduced in studies of EPC mobilization in patients recovering from burns, coronary artery bypass graft surgery, or acute MI [36]. In addition, experiments performed in a mouse bone-marrow transplant model indicated that the incorporation of bone-marrow–derived EPCs into the foci of ocular neovascularization after corneal micropocket surgery was greater in mice with hind-limb ischemia than non-ischemic mice [34]. Thus, the mobilization of bonemarrowderived EPCs appears to be a natural response to tissue ischemia, and the mobilized cells become incorporated into sites of vessel growth.

Per2 has been found a regulator of EPC mobilization induced in hind-limb ischemia [37]. To investigate the reasons for the fewer number of EPCs after MI in per2^−/−^ than WT mice, we studied the mechanisms of EPC mobilization after MI in WT and per2^−/−^ mice. The number of circulating EPCs was linked to alterations in bone-marrow molecular pathways known to be involved in EPC mobilization. Phosphorylated ERK [38]–[41] and Akt [42] signaling plays important roles in bone-marrow EPC mobilization. Here we observed attenuated ERK and Akt signaling in per2^−/−^ mouse bone marrow. The inhibition of ERK and Akt signaling in per2^−/−^ mice led to decreased EPC mobilization.

After MI, bone-marrow derived EPCs incorporated into sites of neovascularization at the border of the infarct area [43] and neoangiogenesis after EPC transplantation improved myocardial blood flow, function and remodeling [44]. Therefore, reduced circulating EPCs early after MI by per2 deficiency likely contributed to decreased capillary density in the peri-infarct area, thus leading to impaired cardiac remodeling and function 28 days post-infarction. In our former investigation [35], DiI-labelled bone marrow derived WT and per2^−/−^ EPCs cultured *in vitro* were injected into mouse myocardium after MI. At 4 weeks, the number of DiI-labelled per2^−/−^ EPCs was lower than that of WT EPCs in the MI myocardium. Decreased number of CD34+ cells in the per2^−/−^ myocardium combined with our former result also led to the weaker angiogenesis.

Bone-marrow–derived adherent cells exhibiting endothelial characteristics such as acetylated low density lipoprotein uptake and lectin binding have been considered cultured EPCs [45]. These cells are positive for CD34, Flk1 and CD31. To study the effect of per2 on EPC function under hypoxia, we cultured EPCs *in vitro* and compared EPC function under hypoxia. Hypoxia for 24 hr increased the migration and tube formation of EPCs as compared with per2^−/−^ EPCs. Our finding agrees with previous investigations [46]–[48]. In the Hoffmann et al. study, hypoxia could increase VEGF-A expression in endothelial cells, which led to enhanced tube formation, and similar results were found in EPCs. In the Kanzler et al. study, hypoxia upregulated CXCR4 and VEGF, thus improving EPC migration and tube formation. In the Yuyu et al. study, hypoxia induced phosphorylation of Akt in EPCs [49]. In the Tobias et al. study, PER2 transcript and protein levels were increased in cardiac tissue of patients with ischemic heart disease. Here, we found increased per2 protein level in EPCs under hypoxia.

Activation of PI3K/Akt signaling was regulated by per2, which was induced by ischemia. However, activation of PI3K/Akt signaling was inhibited in per2^−/−^ EPCs. Therefore, per2 may be a regulator of activation of PI3K/Akt signaling in EPCs under hypoxia. The PI3K/Akt pathway is essential to EPC mobilization, migration, proliferation, and survival [50], so the altered EPC migration and tube formation under hypoxia may depend on activation of PI3K/Akt. *In vitro* hypoxic studies of EPCs are a good explanation for the weak angiogenesis in the per2^−/−^ myocardium after MI.

In conclusion, per2 deficiency aggravates the MI may in part be through decreased EPC bone-marrow mobilization and inhibited EPC function activity.

## Supporting Information

Figure S1 **Western blot analysis (A) of per2 levels.** (B) Quantitative analysis of per2 (** p<0.01 vs WT sham-operated). (C) Representative immunostaining of CD68 (macrophages, 1∶100) in the heart tissue.
(TIF)
