# Correction: Period2 Deficiency Blunts Hypoxia-Induced Mobilization and Function of Endothelial Progenitor Cells

**DOI:** 10.1371/journal.pone.0119196

**Published:** 2015-03-25

**Authors:** The PLOS ONE Staff

The images for Fig. 2B are incorrect. The authors have provided a correct version here.

**Fig 2:**
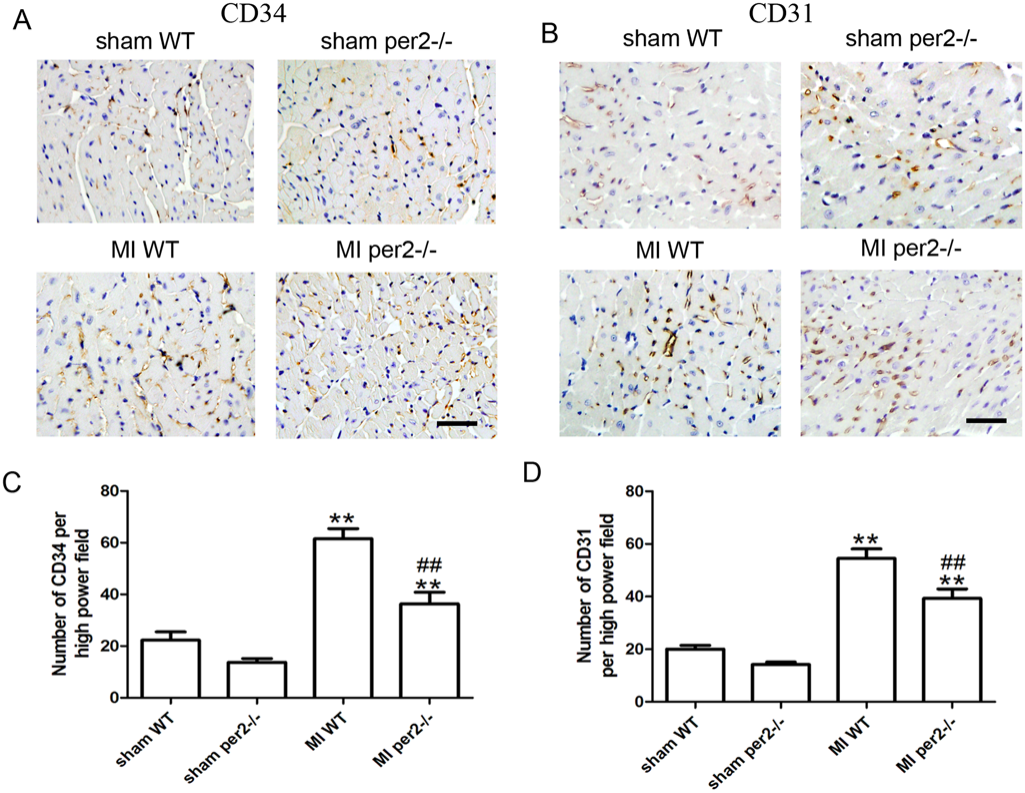
Per2^−/−^ decreased the number of CD34+ progenitors and capillary density in mice. (A) Representative immunostaining of CD34 to identify progenitors and (B) CD31 to identify capillaries. Original magnification: 400×. Quantitative analysis of (C) CD34+ cells and (D) capillary density (** p<0.01 vs sham-operated, ## p<0.01 vs MI WT).

## References

[1] Qin T, Sun Y-Y, Bai W-W, Wang B, Xing Y-F, Yan L, et al (2014) Period2 Deficiency Blunts Hypoxia-Induced Mobilization and Function of Endothelial Progenitor Cells. PLoS ONE 9(9): e108806 doi: 10.1371/journal.pone.0108806 25268972 10.1371/journal.pone.0108806 PMC4182576

