# Supplementary figures and images for: Period2 Deficiency Blunts Hypoxia-Induced Mobilization and Function of Endothelial Progenitor Cells
Source: PLoS One. 2014 Sep 30;9(9):e108806. doi: 10.1371/journal.pone.0108806 (PMC4182576; doi:10.1371/journal.pone.0108806)

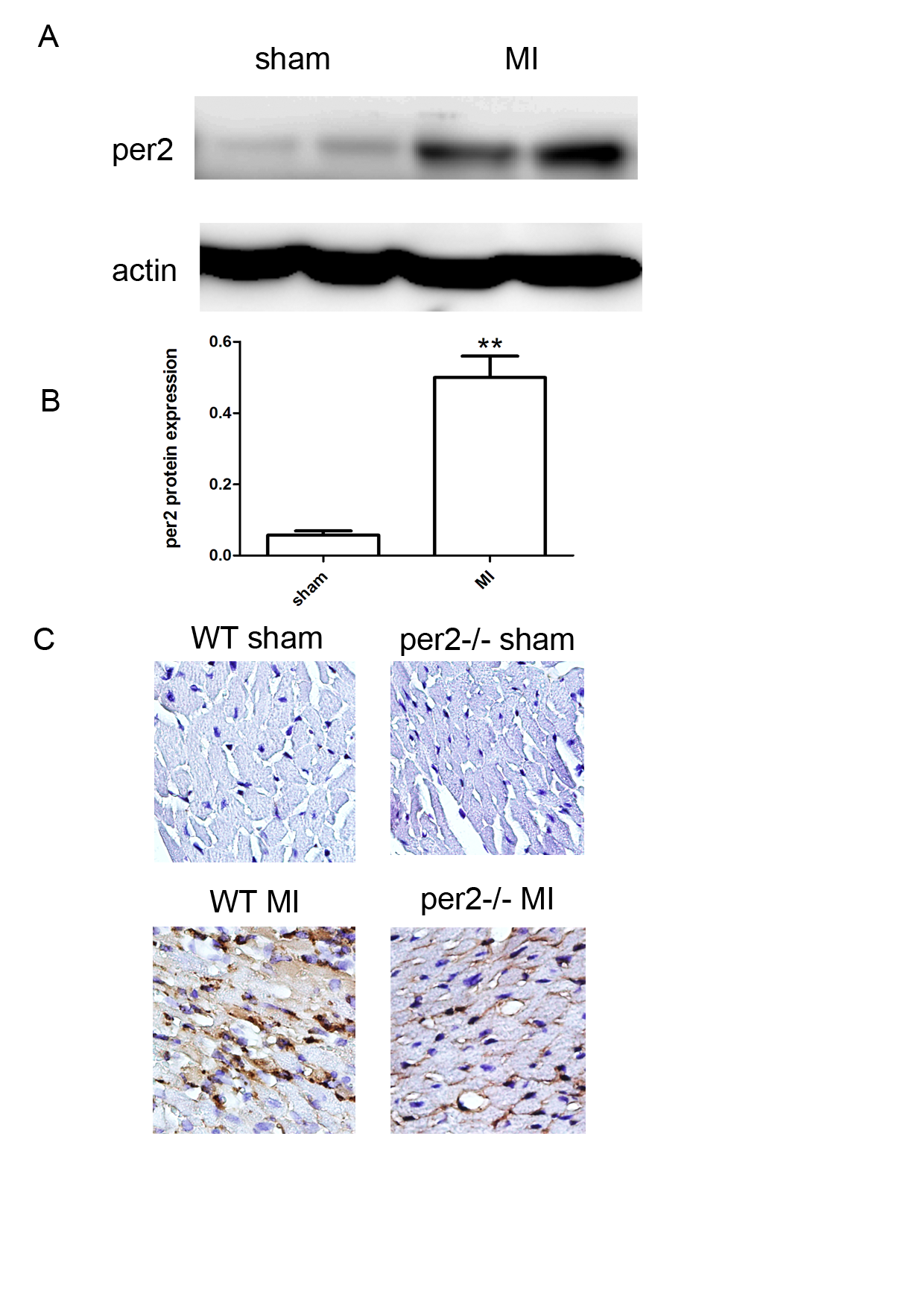

Supplement: Figure S1 — Western blot analysis (A) of per2 levels. (B) Quantitative analysis of per2 (** p<0.01 vs WT sham-operated). (C) Representative immunostaining of CD68 (macrophages, 1∶100) in the heart tissue. (TIF) [file pone.0108806.s001.tif]
